# Development of Criteria for Incorporating Occasionally Consumed Foods into a National Dietary Guideline: A Practical Approach Adapted to the Spanish Population

**DOI:** 10.3390/nu11010058

**Published:** 2018-12-28

**Authors:** Susana Menal-Puey, Iva Marques-Lopes

**Affiliations:** Nutrition and Dietetic Unit, Faculty of Health and Sport Sciences, University of Zaragoza, Plaza Universidad, 3, 22002 Huesca, Spain; imarques@unizar.es

**Keywords:** occasionally consumed foods, dietary guidelines, maximum consumption, quantitative recommendation

## Abstract

Food-based dietary guidelines (FBDGs) offer recommendations that help population to meet nutrient requirements. Most European FBDGs include quantitative information regarding daily and weekly consumed foods, but for occasionally consumed foods, they only recommend limiting their intake, without giving specific advice on portions. As these foods are consumed by the general population as a part of the cultural and culinary tradition of each country, it is important to establish the maximum frequency and the portions that would be acceptable to be included in a healthy eating pattern. This study outlines the methodology to include these foods in a national (Spanish) FBDG. Firstly, commonly consumed foods were selected and grouped, and portions were defined according to their nutritional value, so different foods within a group could be exchanged. Then, macronutrient profiles of occasionally consumed foods were compared to the frequently consumed food groups to determine to what extent they had a similar nutritional content. Finally, some combinations of foods, with or without the inclusion of occasionally consumed groups, were calculated. A maximum number of servings per group was defined according to their energy and nutrient content. Occasionally consumed foods can be included in a healthy diet as long as they are consumed in the small quantities as shown in this study and as long as they replace equivalent portions of other foods of frequent consumption. This new tool shows how to include occasionally consumed foods in a diet while maintaining its overall nutritional quality.

## 1. Introduction

Food-based Dietary Guidelines (FBDGs) are a set of guidance given by governments to help the general population make healthy food choices. The messages and dietary recommendations are designed on the basis of the nutritional objectives of each country and embed scientific knowledge about nutrients, food and health [1,2].

FBDGs should support healthy dietary patterns that meet nutritional and energy needs of a population, maintaining current dietary habits, socioeconomic characteristics and physical and biological environment in a cultural context, in order to be useful in the practical food choices [3]. To improve their effectiveness, FBDGs should indicate frequency and amounts of foods that should be eaten, but this information is not always available.

Different authors have reviewed and compared the food guides of several countries. These studies have shown that, although there are general common messages on healthy eating, the level of specification is different. Worldwide, the majority of countries present a visual food guide with two levels of consumption (frequently and occasionally). In frequently level (daily or weekly), they commonly include foods as whole grains, vegetables and fruits, with broad and unspecific indications (e.g., eat plenty of fruits and vegetables), but others present specific messages with portions and frequencies (e.g., eat five portions of fruits and vegetables), information very useful in planning particular diets [4,5,6].

In the occasionally consumed foods group, most FBDGs consider foods high in salt, sugar and/or unhealthy fats (solid fats, red meats, sugar foods and desserts) and processed (deep fried foods, candies, processed meats and industrial pastries), including simply advising moderation (e.g., limit red meat intakes). Very few refer to the specific quantities or maximum frequency with which they should be eaten. In relation to the European context, Estonia FDBG make a reference to frequency intake of added solid fats (3–5 portions daily, 1 teaspoon (tsp) of margarine or butter, 2 tsp of low-fat margarine). Portugal’s and Turkey’s FDBGs refer to a maximum intake of 20–40 g and 30–40 g/day of sugar, respectively, and Estonia’s FDBG makes reference to other sugar foods (2–4 portions daily, 2 tsp sugar, honey or jam, 2 pieces of cakes, 10 g of chocolate, 150 g of soft-drink) [7]. Moreover, maximum red meat and processed meat intakes are referenced in the Mediterranean diet food guide (<2 and <1 portion/week, respectively) [8].

Although several studies have shown a positive association between consumption of these foods and non-communicable diseases [9,10], they are regularly eaten and can contribute to the overall enjoyment of eating, often in the context of social activities and family or cultural celebrations [11]. Spanish diets are characterized by an inclination toward meat products [12]; Central and Northern European countries show a preference for red meats [13]. In this way, it would be necessary to define recommendations about specific portions for all these foods in order to consider the individual preferences and cultural patterns within a healthy context.

The aim of this research was to define the portion sizes and frequency of consumption of foods containing saturated fat, added sugars and/or sodium which in conjunction with frequent foods do not exceed the maximum intakes of energy and nutrients for the adult population. This research is the first project in Spain (and Europe) to address the inclusion of specific amounts and maximum of servings per day into a FDBG for occasionally consumed foods according energy and concerning nutrients allowance. Due to the lack of this information in other European FBDGs, this pilot approach could be established in other countries using the methodology described.

## 2. Materials and Methods

The research underpinnings of this project had four phases: (1) selection and grouping of foods commonly consumed in Spain and available in national supermarkets; (2) study of nutritional composition in macronutrients and other concern nutrients for each food group; (3) definition of common portions of the selected foods in order to square groups; and (4) determination of the maximum daily servings for each food group in conjunction with the current dietary recommendations of the national guideline.

### 2.1. Phase 1

The foods selected for this work were those most representative of Spanish food habits and available in most of the country’s supermarkets. They were grouped according to the main ingredient and the groups were as follows: sugars, animal fats, fatty meats, and processed foods which included the following subgroups: meat products, fat cheeses, sugar-added milk desserts, confectionary, chips/snacks, and miscellaneous (pre-prepared foods).

### 2.2. Phase 2 and Phase 3

Once preliminary groups were defined, centesimal composition of selected foods were compiled from National Food Composition Database [14] and/or other sources such as values of branded food labels. From these data, the food portions that could be in the same group, without significant differences in macronutrient and other relevant nutrients were determined. In this way, different amounts of each food were introduced in nutritional composition database, and nutrient values were studied. To adjust the weight of each food within the same group, a statistical criterion to design food exchange lists previously published by the authors [15] has been used. The used criteria were as follows (Table 1): For macronutrients, the recommended values of standard deviation (SD) were considered. Once the SD was adjusted, the coefficient of variation (CV) was also studied, aiming for values less than 30%. For groups with higher values of CV, the *z*-value for each food was calculated, establishing as criteria *z* score between ±2, to detect foods with high variations. All the amounts of foods (in grams) were established according to the packing size of foods in market (i.e., 2 small units of pastries) and/or the Spanish household measures (i.e., one teaspoon of butter).

After comparison of macronutrients in each group, other relevant nutrients with high dietary contribution were compared looking for CV values less than 30% [16]. If a CV value was outside limits in a relevant nutrient of a group, the *z* score for each food was calculated identifying those foods responsible for that variation. According to the European Regulation on nutrition-related declarations in foods [17] the criteria for selecting significant nutrients in a food group are as follows: a food group is considered high in sugars when it contains more than 5% of the mean net weight in solid foods, and more than 2.5% in liquid foods. In the same way, the saturated fatty acids (SFAs) content is considered high when they provide more than 10% of the energy value of the food group. For other nutrients of concern, traffic light cutoffs were considered (20% of the daily value) [18]. A food group is high in sodium when it contains more than 480 mg per 100 g, 20% of the European recommended intakes [17], and high in cholesterol when it contained more than 60 mg per 100 g, 20% of American Daily Value [19].

### 2.3. Phase 4

The occasionally consumed foods could be included in a dietary pattern as long as the daily nutritional requirements could be maintained. In this sense, occasional servings were defined in conjunction with current dietary recommendations of the national guideline [20]. The number of servings for each group was defined in the basis of the maximum intakes of nutrients of concern for the adult population. A maximum total fat intake of 30–35% of total caloric value (TCV) and a SFAs intake that did not exceed 10% of TCV were deemed healthy recommendations [21]. Special attention should be given to free sugars, with a maximum daily intake of 5% of TCV [22]. Therefore, a global recommendation of <2000 mg/day of sodium in adults was generally assumed [23]. However, since it is estimated that 20 to 30 percent of the sodium is added with food, its intake recommendation was reduced to 1400 mg. In the absence of European references, the American recommendation of a cholesterol daily intake less than 300 mg/day was taken into account [24]. Because energy and nutrient requirements differ depending on age, sex, and physical activity, a range of servings was suggested for 1500 and 2500 kcal.

## 3. Results

### 3.1. Selection and Grouping of Foods

A total of 81 foods were selected and classified according the follow groups: (a) added sugars: white sugar, brown sugar, cocoa powder, jam, honey, molasses, and syrup; (b) animal fats: butter, pork lard; (c) fatty meats: pork rib, pork leg, pork pancetta, lamb rib, lamb leg, lamb shoulder; (d) fatty meat products: catalan pork sausage, black pudding, mortadella, bacon, chorizo, fuet, longaniza, chistorra, salchichon, sobrassada, salami, canned pork liver; (e) fat cheeses: Roncal, Idiazabal, Zamorano, Manchego, Tetilla, Mahon, Cabrales, Casar, Majorero, Cheddar, Roquefort, Gruyere, Brie, Emmental, Raclette, Edam, Gouda, Camembert, mini portion cheese, sliced to melt cheese; (f) sugar added dairy desserts: egg flan, commercial custard, milk rice, milkshake of cocoa; (g) confectionary: cake, suisse brioche, croissant, ensaimada, cupcake, chocolate cake, digestive biscuit, Marie biscuit, tea cookie, apple pie; (h) chips and snacks: toasted corn, wheat crust, several crackers, corn pufs, popcorn, chips; (i) miscellaneous (pre-prepared foods): Frankfurt sausages, canned meatballs, meat dumplings, breaded pork, chicken croquettes, meat cannelloni with bechamel sauce, meat lasagna, breaded fish, fish croquettes, romana squids, tomato cheese pizza, cheese dumplings, spring rolls.

### 3.2. Establishment of Food Portions and Definition of Definitive Food Groups according to Statistical Criteria

After studying the content of macronutrients in the portions defined, the general food groupings were reorganized according to the aforementioned statistical criteria. In this way, the fat meat product group was grouped with fat cheeses because of the similar energy and macronutrient content. The definitive food groups and subgroups and the portions proposed for the foods of each are presented in Table 2. The food portions were defined in grams and in household measures and were referred to raw and net weight.

From the calibration of the food portions indicated in Table 2, the mean energy, macronutrient, and other nutrient values for each group were obtained. These mean data along with the statistical parameters calculated in this study are shown in Table 3. Those groups with high variability in a relevant nutrient (gray-shaded cells), were analyzed and foods with *z* scores outside limits were identified. Although the *z* score for each one was not shown, the list of foods with *z* scores outside limits in groups with high CV value is presented in Table 4. For example, fatty meat products/cheeses group presented a mean sodium value of 295.7 mg per portion. Nevertheless salami presented a *z*-score >2, which distances it considerably from the group mean.

### 3.3. Determination of Number of Servings per Group

The number of servings for each occasionally consumed group was defined in conjunction with the current dietary recommendations of the national guideline for two levels of total daily calories (1500 and 2500 kcal): 3–6 portions of cereals, bread or tubers, 2 portions of vegetables, 3 portions of fruits, 3–6 portions of vegetal oil, 2 portions of defatted milk and dairy, 1–3 portions of defatted meat, fish, eggs, and legumes [20]. In this way, it was necessary to compile the mean nutritional content of frequently consumed food groups to be compared to the occasionally consumed food groups in order to determine to what extent both groups are equivalent in macronutrients. Table 5, shows the energy and mean nutritional content of both, frequently and occasionally food groups, rounded. The pairing macronutrients of groups were as follows:

#### 3.3.1. Pairing of the Macronutrients of the Occasionally Consumption Group with those of the Groups of Frequently Consumption

Sugars group: An intake of 1 or 2 portions of added sugars could be ingested in a day for 1500 and 2500 kcal, respectively. One more portion would exceed the recommended limit (<5% TCV).

Fats group: Portions defined in animal fats group were similar in fat content to portions of oils and nuts on Spanish food guide, but with 3 g of SFAs more.

Meats and meat products/cheeses: According to macronutrient values, a portion of fatty meats (i.e., pork or lamb) could be interchangeable with a portion of lean meats (i.e., turkey, chicken, veal) plus one portion of vegetables oils or nuts. In the same way, a portion of fatty meat products (i.e., bacon, cheese, salami, sobrassada) could be interchangeable with a half portion of lean meats if daily oils or nuts portions are adjusted. In both cases, a more saturated fat profile would be ingested. It is necessary to point out that portions of lean meat products established (30 g) could be similar to a half portion of lean meat (30 g), in order to choose them in diets (i.e., cooked ham, cured ham, cured pork loin).

Milk and dairy: Macronutrient content of sugar added dairy desserts could be similar to defatted milk and dairy plus 20 g of extra added sugar and 46 mg of extra cholesterol.

Grains, potatoes, and derivatives: A portion of confectionary or chips/snacks could be interchangeable with half portion of grains/potatoes plus a portion of vegetable oils or nuts. In confectionary group, an extra consumption of sugars was detected (10 g, 2 dessert spoons). These changes assumed a more saturated fatty acid profile and an intake of 61 mg of cholesterol for the confectionary case. An extra intake of sodium should be considered (203 mg and 449 mg, respectively).

Miscellaneous (pre-prepared foods): Comparing their macronutrient composition with food groups of frequent consumption, they are similar to one portion of lean meats plus one portion of fats plus an extra carbohydrates intake (10 g, approx. 40 extra calories per interchange). This could be because these foods are generally manufactured with fatty meats with flours in batters or ligands. An extra intake of sodium should be considered (496 mg).

Taking into account these values, different combinations of foods were calibrated. Firstly, adapted from the Spanish dietary guidelines, 3 or 6 servings of grains, bread and potatoes, 2 servings of vegetables, 3 servings of fruits, 3 or 5 servings of vegetable oils with at less 1 serving of nuts, 2 servings of milk and dairy and 1 or 3 servings of lean meats, fish, eggs or legumes, were assigned for the two energy patterns, respectively. Afterward, different numbers of the frequently consumption food portions were interchanged with occasionally consumption foods and values of nutrients of concern were studied, to verify the maximum number of servings of occasionally consumed foods per day for each group. The different combinations of servings and the nutritional contribution for the two levels of energy intake are shown in Table 6 and Table 7. 

#### 3.3.2. Maximum Number of Occasionally Consumed Food Portions Per Day for Each Group, Independently

1. Added sugars group: An intake of 1 or 2 portions of added sugars could be ingested in a day for 1500 and 2500 kcal, respectively. One more portion would exceed the recommended limit (<5% TCV).

2. Animal fats: In both caloric patterns, it could be possible to interchange 1 portion of vegetable oils for animal fats without altering the fat profile, but it would be necessary to intake 1 or 2 portions of n-3 rich fats in order to get an adequate PUFAs intake.

3. Fatty meats and meat products/cheese: In the 1500 caloric pattern, an intake of 1.5 portions (90 g) of lean meats has been assigned per day. This amount (90 g) could be interchanged with fatty meats without exceeding the maximum SFAs intake (10% TCV). In the 2500 caloric pattern, up to 2 portions of fatty meats (120 g) could be ingested. When it was required, a portion of lean meat products (i.e., cooked ham) or fatty meat products (i.e., chorizo) could be chosen instead of half portion of lean meat, with the corresponding fat adjustments for the second option. For example, 1 portion of cured ham (30 g) can be chosen instead of half portion of poultry (30 g). If fatty meat products are chosen (i.e., chorizo), the daily fat should be adjusted (half portion less). For 2500 kcal of consumption, 2 portions of fatty meat products could be ingested without exceeding the saturated fats and the sodium intakes.

4. Sugar added dairy desserts: For both caloric patterns, it could be possible to interchange 1 portion of the lean milk and dairy group for 1 of sugar added dairy desserts without exceeding the recommended limit of sugars (<5% TCV), 1 more portion would excess this limit.

5. Confectionary: For both caloric patterns, when 1 portion was interchangeable for cereals/potatoes and fats, the SFAs, cholesterol and sodium intakes were within the limits (<10% TCV, <3000 mg, <1400 mg, respectively). Despite the extra sugar intake involved in this exchange, it is not above the healthy limits (<5% TCV). When 2 servings were selected in 2500 kcal pattern, the fat profile and cholesterol intake was not exceeded (10% TCV in saturated fats; 267 mg, respectively).

6. Snacks: For both the 1500 and 2500 kcal patterns, up to 1 serving of snacks could be chosen without exceeding the intake of 1400 mg of sodium. In both cases, 2 servings could reach the limit of sodium intake, but it must be noted that the calibration of basic cereal group was carried out with low-salt frequent foods.

7. Miscellaneous (pre-prepared foods): Only 1 portion of this food group was possible to include, because of the high sodium consumption (>1400 mg/day).

#### 3.3.3. Possible Daily Simultaneous Consumption of Occasionally Consumed Food Groups

Taking into account the concerning nutrients in each group, possible combinations of occasional groups has been considered. In relation to sodium intake, fatty meat products or cheeses, confectionary, chips/snacks, and miscellaneous were the most contributing groups. Added sugars, sugar-added dairy desserts or confectionary groups could involve a high intake of sugars. In the same way, for SFAs, animal fats, fatty meats, fatty meat products or cheeses, sugar added dairy desserts, confectionary, chips/snacks and miscellaneous should be controlled. Table 8 shows the possible simultaneous consumption of groups for the two aforementioned caloric patterns.

As can be seen, there are limitations when these groups are consumed in the same day. For the 1500 caloric pattern, added sugars should not be combined with sugar added dairy desserts or confectionary although a single sugar is permitted; however, they could be combined for higher energy intakes. Likewise, in order to control saturated fat intake, only one daily group of animal fats, fatty meats, fat products/cheeses, sugar added dairy desserts, confectionary, chips/snacks or miscellaneous could be ingested for a 1500 kcal diet, nevertheless two of them could be present for the 2500 kcal pattern as long as the sodium intake is not excessive (for example, chips/snacks plus miscellaneous should be avoided).

#### 3.3.4. Dietary Recommendations

Applying the previous information regarding food groups, servings and nutritional content, the dietary recommendations for occasionally consumed foods are listed in Table 9. For a better practical application, they were grouped according the concerning nutrient and recommendations of simultaneous intake are included.

As can be seen, it is possible to consume foods with a considerable amount of sugars, SFAs, and sodium in a healthy context of nutrients, nevertheless, some of the foods included in the SFAs groups (fatty meat products) could contain other chemical components that could produce adverse effects for humans. The presence of n-nitroso compounds (NOCs) formed in meat products cured with nitrite and/or nitrate or in the human digestive system from the nitrates presented in meat products are the main mechanisms involved in this risk [25]. In this regard, an acceptable daily intake (ADI) for nitrates of 3.7 mg/kg body weight per day was defined, i.e., 222 mg/day for a mean body weight of 60 kg [26].

Taking into account the maximum amount of nitrate that could be found in meat products in Europe (150 mg/kg of product) [27], it can be calculated that the maximum estimated daily intake (EDI) with 2 portions (60 g), the maximum amount recommended in this guide, would be around 9 mg, 4% of the ADI.

## 4. Discussion

Worldwide, a large number of dietary guidelines to help population make healthy food choices are available. Most of them include concise messages about frequency and amounts of foods considered as frequent consumption in the diet, but very few include information about occasionally consumed foods. This practical approach includes specific quantities and maximum frequency of consumption of these food groups in order to include them in menu planning, when possible.

With regard to food groups, while most food guides included groups generally recognized for contributing fats or a particular type of fat, carbohydrate, protein, or vitamin and mineral, none of the current guides provide references to groups taking into account nutrients of concern such as sodium, cholesterol, sugars, or saturated fats. This tool offers a wide number of foods typically consumed in Spain included in groups in terms not only of carbohydrate, protein, and fat, content but also other nutrients of interest, and a list of foods marked as exceptions in the contribution of any of these nutrients (upward and downward). Nutrition and dietetics and nutrition practitioners could make food choices according to particular preferences without significant variations in nutrient intakes, but they should take into account the possible deviation with specific foods per group, for example higher sodium content in salami or lower in cake. Also, all the portion sizes incorporated in this research take into account the packing size of foods in market or to the Spanish household measures. This is a beneficial characteristic compared to other guides because it could assist consumers in the portion size measures as an effective strategy for energy control [28].

With regard to frequency of consumption, this research offers a new methodology to incorporate these foods into one´s diet. Occasionally consumed food servings could be included instead of other basic groups in terms of macronutrient content, but if professionals add them as “extra foods”, high caloric diets could be reached. In Australia, a food tool-kit was developed to calculate the maximum number of occasionally consumed food servings that could be included in a diet as extra energy in active individuals, but not in the low-calorie pattern [11]. To the author´s knowledge, this is the first tool to present the combination of portions of frequently consumed foods to fit each portion of occasionally consumed foods, in order to adjust macronutrient intakes and to allow for the consumption by anyone (different caloric intakes).

This research not only determines the interchanges of frequently consumed foods with occasionally consumed foods, but also provides a range of servings per day for each occasionally consumption group according the energy and the recommendations for nutrients of concern. If dietetics and nutrition practitioners adjust the diet to lower portions, 1500 kilocalories should be reached in a healthy way and up to 2500 kcal per day if larger portions are given.

This is the first practical translation of guidelines of limited consumption of Spanish dietary guidelines [20] to a quantitative recommendation, and to include rules to the simultaneous intake of different groups in the same day. In this sense, professionals could opt to combine groups in higher caloric intakes as long as the recommended maximum intakes are met (for example, one portion of sugar-added milk desserts plus one portion of fatty meat products).

Finally, it is necessary to note that foods listed in this database are generic, and it is possible that other brands present different nutritional profiles. Therefore, the authors recommend the use of nutrition facts from food labels to check for possible deviation from the group, and to select those low in salt.

## 5. Conclusions

This manuscript described the methodology to include in a FBDG those foods that should be occasionally consumed, with the practical application to the Spanish population. An adequate selection and classification of foods, with adjusted portions can make possible the inclusion of them in a meal plan adjusted for varying needs and preferences. It is necessary to consider different rations to cover the energetic recommendations of the population in question.

This work exposes a methodology that allows designing dietary recommendations for the inclusion of occasionally consumed foods in a national dietary guideline. To be able to serve as a tool for health professionals and for the population it would be necessary to develop appropriate iconographic formats for their use.

## Figures and Tables

**Table 1:** Statistical criteria proposed for the definition of the food portions in each group.

	Standard Deviation (SD)	Coefficient of Variation (CV)	*z* Values for Each Food
Energy	± 20 kcal	30%	± 2
Carbohydrate	± 5 g		
Fat	± 2 g		
Protein	± 3 g		

**Table 2:** Food groups and serving sizes proposed to the occasional foods.

Group or Subgroup	Foods	Raw and Net Weight, g (Household Measure)
1. Added Sugars	White and brown sugar Cocoa powder Jam Honey, molasses, syrup	10 (2 teaspoons) 10 (2 teaspoons) 15 (1 heaping teaspoon) 15 (1 tablespoon)
2. Animal Fats	Butter Pork lard	10 (2 teaspoons) 10 (2 teaspoons)
3. Fatty Meats	Pork rib Pork leg Pork pancetta Lamb rib Lamb leg Lamb shoulder	60 (2 pieces) 60 (2 medium chops) 50 (2 medium slices) 60 (3 pieces) 60 (2 medium chops) 60 (1 portion)
4. Fatty Meat Products and Cheeses	Catalan pork sausage Black pudding Mortadella Bacon Chorizo Fuet Longaniza Chistorra Salchichon Sobrassada Salami Canned pork liver Cheeses of different origins Mini portion cheese	40 (4 medium slices) 30 (1 medium slice) 30 (3 thin slices) 30 (3 thin slices) 30 (6 thin slices) 25 (6 thin slices) 30 (6 thin slices) 30 (1 portion of 15 cm) 30 (6 thin slices) 20 (1 portion) 30 (2 thin slices) 20 (1 portion) 30 (1 thin slice) 30 (2 pieces)
	Slice to melt cheese	40 (2 regular slices)
5. Sugar Added Dairydesserts	Egg flan Custard Milk rice Milkshake of cocoa	110 (1 commercial recipe) 125 (1 commercial recipe) 140 (1 commercial recipe) 200 (1 commercial recipe)
6. Confectionary	Cake Suisse brioche Croissant Ensaimada Cupcake Chocolate cake Digestive biscuit Marie biscuit Tea cookie Apple pie	60 (2 thin portions) 60 (2 units) 60 (4 small units) 60 (2 small units) 60 (2 units) 60 (2 thin portions) 60 (4 units) 50 (6 units) 60 (4 units) 80 (2 thin portions)
7. Chips and Snacks	Toasted corn Wheat crust Crackers Crackers with cheese Corn pufs Popcorn Chips	40 (1 small bag) 40 (½ small bag) 48 (4 units) 48 (4 units) 40 (1 small bag) 40 (1 small bowl) 40 (1 small bag)
8. Miscellaneous	Frankfurt sausages Canned meatballs Meat dumplings Breaded pork Chicken croquettes Meat cannelloni with bechamel sauce Meat lasagna Breaded fish Fish croquettes Romana squids Tomato cheese pizza Cheese dumplings Spring rolls	80 (2 pieces) 120 (4 pieces) 60 (2 pieces) 75 (1 piece) 120 (3 pieces) 120 (2 pieces) 150 (1 portion) 150 (3 pieces) 120 (3 pieces) 120 (6pieces) 100 (2 portions) 60 (2 pieces) 120 (2 pieces)

**Table 3:** Mean nutrient content, standard deviation (SD) and coefficient of variation (CV) of food groups based on servings of foods.

Group or Subgroup	Energy (kcal) Mean ± SD (CV)	Protein (g) Mean ± SD (CV)	Fats (g) Mean ± SD (CV)	SFA ^a^ (g) Mean ± SD (CV)	MFA ^b^ (g) Mean ± SD (CV)	PFA ^c^ (g) Mean ± SD (CV)	Cholesterol (mg) Mean ± SD (CV)	CHO ^d^ (g) Mean ± SD (CV)	Sugar (g) Mean ± SD (CV)	Na (mg) Mean ± SD (CV)
1. Added sugars	41 ± 4 (9.7)	0	0	0	0	0	0	0	9.6 ± 1.1 (11.4)	0
2. Animal fats	82.2 ± 9.8 (11.9)	0	9.1 ± 1.1 (12.2)	4.7 ± 0.6 (12.9)	3.3 ± 1.2 (35.2)	0.6 ± 0.5 (77.4)	10.1 ± 0.8 (7.7)	0	0	1.2 ± 1.4 (117.9)
3. Fatty meats	147.3 ± 14.8 (10.1)	10 ± 2.5 (17.0)	11.9 ± 1.9 (16)	5 ± 1.1 (22.9)	5 ± 1.2 (24)	1.1 ± 0.4 (38.6)	41.2 ± 8.1 (19.6)	0	0	43.3 ± 4.4 (10)
4. Fatty meat products/cheeses	113.5 ± 15.4 (13.6)	6.2 ± 1.6 (25.4)	9.7 ± 1.8 (18)	5.1 ± 1.4 (28.3)	3.4 ± 1.4 (41.1)	0.7 ± 0.5 (70.3)	27 ± 5.7 (21.1)	0.7 ± 0.3 (41.6)	0	295.7 ± 146.8 (49.7)
5. Sugar-added dairy desserts	146.7 ± 14.3 (9.7)	5.1 ± 0.7 (12.8)	4.1 ± 1.2 (29.3)	2.3 ± 0.9 (39.1)	1.3 ± 0.7 (50.8)	0.3 ± 0.2 (83.4)	53.2 ± 72.6 (136.5)	2.3 ± 2.7 (115)	20.3 ± 1.7 (8.3)	76.1 ± 19.2 (25.2)
6. Confectionary	250.4 ± 23.3 (9.3)	3.9 ± 0.9 (21.7)	12.8 ± 2.7 (21.0)	6.5 ± 1.4 (22.0)	4.4 ± 1.6 (36.1)	1.1 ± 1 (90.0)	60.7 ± 32.8 (54.0)	19.8 ± 6.3 (31.6)	10.2 ± 4.9 (48.3)	207.2 ± 107.3 (51.8)
7. Chips/snacks	209.1 ± 13.7 (6.6)	3.9 ± 1.1 (28.2)	12 ± 1.3 (10.9)	4 ± 1.5 (37.9)	4.5 ± 1.3 (28.8)	2.9 ± 1.7 (57)	14.4 ± 4.4 (30.5)	20.1 ± 0.9 (4.5)	1.4 ± 1.4 100	453.3 ± 138.4 (30.5)
8. Miscellaneous	206.7 ± 25.1 (12.1)	10.5 ± 3.9 (37.2)	12.1 ± 4.3 (35.5)	4.4 ± 2.5 (57.3)	4.5 ± 2.2 (48.8)	2.4 ± 1.2 (50.4)	57.1 ± 43.6 (76.3)	12.8 ± 6.8 (53.1)	1.1 ± 1.2 (108.9)	536.9 ± 264.1 (49.2)

Gray-shaded cells mark a relevant nutritional contribution in groups or subgroups according to European regulation and other criteria. ^a^ SFA: saturated fatty acid. ^b^ MUFA: monounsaturated fatty acid. ^c^ PUFA: polyunsaturated fatty acid. ^d^ CHO: carbohydrates.

**Table 4:** Foods with *z*-values outside limits in relevant nutrients of the groups. A frequent choice of these foods may upset this nutrient intake.

Group/Subgroup	Nutrients with High Variability in Group	Foods with *z* Score > 2	Foods with *z* Score < 2
Fatty meat products/cheeses	Sodium	Salami	-
Sugar added dairy desserts	Cholesterol	Egg flan	-
Confectionary	Cholesterol Sodium	Swiss brioche -	- Cake
Chips and snacks	SFAs Sodium	Crackers -	- Corn puffs
Miscellaneous	Cholesterol Sodium	Romana squids Canned meatballs	- -

**Table 5:** Mean energy and nutritional content of frequently and occasionally food groups defined in this research, subjected to rounding.

Food Groups or Subgroups (Net Weight Portion, g)	Energy (kcal)	Protein (g)	Fats (g)	SFA ^a^ (g)	MFA ^b^ (g)	PFA ^c^ (g)	Cholesterol (mg)	CHO ^d^ (g)	Sugar (g)	Na (mg)
SUGARS GROUP										
1. Added Sugars (10 g)	41	0	0	0	0	0	0	0	10	0
FATS GROUP										
2. Animal Fats (10 g)	90	0	9	5	3	1	10	0	0	1
Vegetal Oils (10 g)	90	0	10	2	7	1	0	0	0	0
Nuts (16 g)	90	0	10	2	4	4	0	0	0	30
MEATS AND MEAT PRODUCTS										
3. Fatty Meats (50–60 g)	147	10	12	5	5	1	41	0	0	43
Lean Meats (60 g)	66	12	2	0	1	1	43	0	0	41
4. Fatty Meat Products/Cheeses (30 g)	114	6	10	5	3	1	27	1	0	296
Lean Meat Products (30 g)	41	7	1.5	0.5	0.7	0.2	18	0	0	296
MILK AND DAIRY										
5. Sugar Added Dairy Desserts (110–200 g)	147	5	4	2	1	0	53	2	20	76
Deffated Milk and Dairy (200–250 g)	72	7	1.5	1	0.5	0	7	6	2	80
GRAINS, POTATOES AND DERIVATIVES										
6. Confectionary (50–60 g)	250	4	13	7	4	1	61	20	10	207
7. Chips and Snacks (40–48 g)	209	4	12	4	5	3	14	20	1	453
Grains and Potatoes (30–200 g)	210	6	2	0	0	0	0	42	0	4 ^e^
OTHERS										
8. Miscellaneous (60–150 g)	207	11	12	4	5	2	57	13	1	537

Gray-shaded cells mark a relevant nutritional contribution in groups or subgroups according to European regulation and other criteria. ^a^ SFA: saturated fatty acid. ^b^ MUFA: monounsaturated fatty acid. ^c^ PUFA: polyunsaturated fatty acid. ^d^ CHO: carbohydrates. ^e^ Breads low in salt.

**Table 6:** Suggested number of servings for occasional foods at 1500 kcal.

FOOD GROUPS	Basic Foods	With Occasional Foods												
		Added Sugars		Animal Fats		Fatty Meat	Fatty Products/Cheese		Milk Desserts	Confectionary		Chips/Snacks		Miscellaneous
Grains and Potatoes	3	3		3		3	3		3	2.5	2	2.5	2	3
Vegetables	2	2		2		2	2		2	2		2		2
Fruits	3	3		3		3	3		3	3		3		3
Vegetable Fats At Least 1 Reach in w-3	3 1	3 1		2 1	1 1	1.5 1	2 1	1 1	3 1	2 1	1 1	2 1	1 1	2 1
Defatted Milk/Dairy	2	2		2		2	2		1	2		2		2
Lean Meat	1.5	1.5		1.5		0	1	0.5	1.5	1.5		1.5		0.5
Added Sugars	0	1	2	0		0	0		0	0		0		0
Animal Fats	0	0		1	2	0	0		0	0		0		0
Fatty Meats	0	0		0		1.5	0		0	0		0		0
Fatty Products/Cheeses	0	0		0		0	1	2	0	0		0		0
Sugar Added Dairy Desserts	0	0		0		0	0		1	0		0		0
Confectionary	0	0		0		0	0		0	1	2	0		0
Chips and Snacks	0	0		0		0	0		0	0		1	2	0
Miscellaneous	0	0		0		0	0		0	0		0		1
Energy (kcal) Protein (% CTV) Carbohydrate(% CTV) Fat (% CTV) PUFAs (% CTV) SFAs (% CTV) MFAs (% CTV) Cholesterol (mg) Sugars (% CTV) Sodium (mg)	1490 15 52 33 6 6 15 78 0 355	1530 ^a^ 15 51 31 6 6 15 78 3 355	1570 ^a^ 15 49 31 6 6 15 78 5 355	1482 15 53 32 6 8 13 90 0 356	1474 15 53 32 6 11 10 100 0 357	1480 15 52 33 6 9 13 76 0 358	1480 15 52 33 6 9 13 85 0 630	1471 15 53 32 6 11 10 91 0 905	1565 ^b^ 14 48 32 6 7 15 126 5 351	1545 ^c^ 15 50 32 6 9 14 140 3 560	1600 ^c^ 15 48 32 5 12 11 201 5 765	1504 15 52 33 7 8 14 94 0 806	1518 15 33 51 8 9 13 107 1 1257	1540 ^d^ 15 54 31 6 7 13 94 1 850

^a^ The extra sugar intake of added sugars (10 g, 40 extra calories per interchange). ^b^ The extra sugar intake of sugar added dairy desserts versus defatted dairy (20 g, approx. 80 extra calories per interchange). ^c^ The extra sugar intake of confectionary versus cereals (10 g, approx. 40 extra calories per interchange). ^d^ The extra CHO intake of miscellaneous versus other protein foods of the flours in batters or ligands (10 g, approx. 40 extra calories per interchange).

**Table 7:** Suggested number of servings for occasional foods at 2500 kcal.

FOOD GROUPS	Basic Foods	With Occasional Foods															
		Added Sugars		Animal Fats		Fatty Meats		Fatty Products/Cheese		Dairy Desserts		Confectionary		Chips/Snacks		Miscellaneous	
Grains and Potatoes	6	6		6		6		6		6		5.5	5	5.5	5	6	
Vegetables	2	2		2		2		2		2		2		2		2	
Fruits	3	3		3		3		3		3		3		3		3	
Vegetable Fats At Least 1 Reach in w-3	5 2	5 2		4 2	3 2	4 2	3 2	4 2	3 2	5 2		4 2	3 2	4 2	3 2	4 2	3 2
Defatted Milk/Dairy	2	2		2		2		2		1	0	2		2		2	
Lean Meat	3	3		3		2	1	2.5	2	3		3		3		2	1
Added Sugars	0	1	2	0		0		0		0		0		0		0	
Animal Fats	0	0		1	2	0		0		0		0		0		0	
Fatty Meats	0	0		0		1	2	0		0		0		0		0	
Fatty Products/Cheeses	0	0		0		0		1	2	0		0		0		0	
Sugar Added Dairy Desserts	0	0		0		0		0		1	2	0		0		0	
Confectionary	0	0		0		0		0		0		1	2	0		0	
Chips and Snacks	0	0		0		0		0		0		0		1	2	0	
Miscellaneous	0	0		0		0		0		0		0		0		1	2
Energy (kcal) Protein (% CTV) Carbohydrates (% CTV) Fat (% CTV) PUFAs (% CTV) SFAs (% CTV) MFAs (% CTV) Cholesterol (mg) Sugars (% CTV) Sodium (mg)	2488 15 52 33 6 7 15 145 0 458	2528 ^a^ 15 50 33 6 7 15 145 2 458	2568 ^a^ 14 50 33 6 7 15 145 3 458	2480 15 52 33 6 8 14 155 0 459	2472 15 52 33 6 10 13 165 0 460	2479 15 52 33 7 8 15 143 0 460	2470 14 52 34 6 9 13 141 0 462	2479 15 52 33 6 8 14 150 0 733	2470 15 52 33 6 9 13 156 0 1009	2563 ^b^ 14 49 34 6 7 15 191 3 454	2638 ^b^ 14 47 33 6 7 15 237 6 450	2543 ^c^ 15 50 33 6 8 14 206 2 663	2598 ^c^ 15 49 34 6 10 13 267 3 868	2502 15 51 34 7 8 15 159 0 909	2516 15 51 34 8 8 14 173 0 1361	2540 14 53 33 6 7 14 159 0 954	2590 ^d^ 14 32 54 6 7 13 173 0 1450

^a^ The extra sugar intake of added sugars (10 g, 40 extra calories per interchange). ^b^ The extra sugar intake of sugar added dairy desserts versus defatted dairy (20 g, approx. 80 extra calories per interchange). ^c^ The extra sugar intake of confectionary versus cereals (10 g, approx. 40 extra calories per interchange). ^d^ The extra CHO intake of miscellaneous versus other protein foods of the flours in batters or ligands (10 g, approx. 40 extra calories per interchange).

**Table 8:** Food groups with control in the simultaneous consumption in a day and concerning nutrient per group. Guides for two caloric patterns.

Key Nutrient	Food Groups	1500 kcal	2500 kcal
**Added Sugars**	Added sugars Sugar added dairy desserts Confectionary	Only one group per day	Until 2 groups per day (with limitations explained in Table 9)
**SFAs**	Animal fats Fatty meats Fatty meat products/cheeses Sugar added dairy desserts Confectionary Chips/snacks Miscellaneous	Only one group per day	Until 2 groups *per* day (with limitations explained in Table 9)
**Sodium**	Fatty meat products/cheeses Confectionary Chips/snacks Miscellaneous	Only one group per day	Only one group per day

**Table 9:** Dietary guidelines for occasional food groups according the concerning nutrient per group.

Food Group	Key Nutrients	Exchanges with Basic Food Groups
1. Added Sugars	Sugars	It is possible an extra consumption of sugars in conjunction with the frequent foods in a diet.
2. Animal Fats	SFAs	1 portion of the group counts as 1 portion of vegetable oils. It should be necessary to intake, at least, 1 daily portion rich in omega-3.
3. Fatty Meats	SFAs	1 portion of the group counts as 1 portion of lean meats plus 1 portion of vegetables oils.
4. Fatty Meat Products/Cheeses	SFAs and sodium	1 portion of the group counts as half portion of lean meats plus 1 portion of oils.
5. Sugar Added Dairy Desserts	Sugars and SFAs	1 portion of the group counts as 1 defatted milk and dairy portion with 2 portions of sugars.
6. Confectionary	Sugars, SFAs and sodium	1 portion counts as half portion of grains/potatoes plus 1 portion of vegetable oils with an extra sugar intake.
7. Chips/Snacks	SFAs and sodium	1 portion should be interchangeable for half portion of grains/potatoes plus 1 portion of vegetable oils.
8. Miscellaneous	SFAs and sodium	1 portion counts as 1 portion of lean meats with 1 portion of fats plus an extra carbohydrates intake.
**Food Groups by Key Nutrient**	**Groups per day**	**Practical Application**
**Rich in Sugar Groups** Added Sugars Sugar Added Milk Desserts Confectionary	1–2	For 1500 kcal, you could choose only 1 of the groups in the same day. For example, if you take 1 added sugars portion, sugared desserts or confectionary should not be selected in the same day. For 2500 kcal, you could intake 2 groups. You could mix 1 portion of added sugars with 1 of sugar added dairy desserts or confectionary, or 2 of added sugars or confectionary: Milk desserts should not be repeated.
**Rich in SFAs Group** Animal Fats Fatty Meats Sugar Added Milk Desserts Confectionary Fatty Meat Products Chips/Snacks (sodium) Miscellaneous (sodium)	1–2	For 1500 kcal you could choose only 1 of the groups in the same day. For 2500 kcal, 2 groups could be present in the same day as long as the sodium intake should not be excessive. It should be possible to combine fatty meats or animal fats groups or fatty meat products (low in salt) with the others or repeat them, but the rich in sodium groups should not be combined or repeated (only 1 portion of them). An alternate selection of groups should be made. Be careful with food groups with 2 key nutrients (sugar added dairy desserts and confectionary), they counts as portions of the 2 groups defined (i.e.,: if you take 1 portion of sugared desserts, it counts as portion of rich in sugar plus rich in SFAs). They should not be repeated.
